# Effect of counselling intervention on stress and expressed emotions among family caregivers of children with autism

**DOI:** 10.25122/jml-2023-0261

**Published:** 2023-11

**Authors:** Hend Karem Mahmoud, Ghada Mohammed Mourad, Rania Abdel-Hamid Zaki, Hoda Sayed Mohammed

**Affiliations:** 1 Psychiatric/Mental Health Nursing Department, Faculty of Nursing, Ain Shams University, Cairo, Egypt

**Keywords:** stress, expressed emotions, family caregivers, children with autism

## Abstract

Parenting a child with autism represents an extraordinary challenge for families, resulting in prominent levels of stress and burden that subsequently affect their expressed emotions. This study aimed to evaluate the effect of counseling intervention on stress and expressed emotions among family caregivers of children with autism. The research used a pre-post interventional design, to evaluate 40 family caregivers of children with autism. The interviewing questionnaire assessed socio-demographic data, expressed emotions, and parenting stress, revealing that 57.5% of the evaluated family caregivers had severe stress pre-counseling, compared to 25% post-counseling intervention. Additionally, 80% of them had elevated levels of expressed emotions pre-counseling, compared to 32.5% during the post-counseling intervention. Most family caregivers of children with autism experienced significant levels of expressed emotions, and over half of them had severe levels of stress. However, these levels decreased following the implementation of the counseling interventions. Furthermore, there were highly statistically significant correlations between the total levels of expressed emotions and total stress levels among family caregivers before and after the implementation of the counseling intervention.

## INTRODUCTION

Autism Spectrum Disorder (ASD) is a lifelong neurodevelopmental disorder characterized by difficulties in communication and social interaction, as well as repetitive patterns of behavior, interests, or activities that occur during the early developmental period. ASD is the primary cause of disability in children under the age of five, and it is two to five times more common in the male gender [1].

Also, children with ASD often struggle with daily tasks related to self-care and functional independence. ASD affects around 1–1.5% of the global population, highlighting its significant impact [2]. A prevalence study conducted in Giza from 2019 to August 2022 showed that 3,355 children visited a special needs clinic, indicating the prevalence of ASD in that population [3].

Stress is a natural human response that stimulates us to deal with risks and challenges in our lives [4]. Caregivers of children with ASD may experience stress related to the illness, which is characterized by fatigue, anger, guilt, and other negative feelings associated with caring for a chronically ill and dependent child [5]. Additionally, ASD affects various aspects of life and contributes to elevated levels of expressed emotions. Therefore, family caregivers need counseling intervention to manage their stress.

Expressed emotions (EE) reflect the quality of the family atmosphere and are reflected in the attitudes and communication styles of relatives towards an individual. EE can serve as an indicator of the emotional climate experienced by family members, encompassing criticism, hostility, emotional over-involvement, positive remarks, and warmth [6]. The levels of EE in families with children with ASD have been linked to the behavioral problems of children, with negative dimensions of EE being associated with increased maladaptive behavior and decreased social interaction and communication. In contrast, positive dimensions of EE are associated with increased social interaction and communication and decreased maladaptive behavior [7].

Applied counseling interventions to reduce stress and expressed emotions for families who care for children with autism are needed. However, there is a dearth of literature examining the presence or absence of counseling intervention among family caregivers of children with autism. Consequently, caregivers require knowledge, skills, and proper judgment to care for children. As a result, they need counseling interventions that comprise systemic, didactic-psychotherapeutic interventions that are adequate for providing information about the illness and its treatment, to be able to cope with the burden of care.

Providing counseling interventions to family caregivers is crucial in managing the day-to-day lifestyle of children with autism. Nurses are well-positioned to provide emotional support and guidance during this challenging situation, making their involvement in counseling interventions essential [4]. The aim of this study is to implement counseling interventions to reduce stress and expressed emotions among family caregivers of children with autism.

The research hypothesis is that counseling interventions have a positive effect on managing stress and reducing expressed emotions among family caregivers of children with autism.

## MATERIAL AND METHODS

A pre-post interventional study was conducted in the outpatient clinic for autism at the pediatric hospital affiliated with Ain Shams University in Cairo, Egypt. The sample consisted of 40 family caregivers of children with autism who met the following inclusion criteria: children had to be 3–12 years old, with a diagnosis of autism, both genders, and with no other neurological or psychiatric disorder or handicap. The criteria for inclusion for family caregivers of children with autism are family members who provide direct care to the child, of both genders, with an absence of any psychiatric disease and willingness to participate in the study.

### Sample size

The sample size was calculated based on a study conducted by Mirzaei *et al*. [8]. By estimating an effect size of 0.51, based on the mean of expressed emotion at pre-intervention, the mean and standard deviation were 37.26±3.44, compared to 58.11±3.65 post-intervention, with a statistical power of 95%, level of confidence (1-Alpha Error): 95%, Alpha 0.05, Beta 0.1. The needed sample size was thirty-six caregivers, considering a 10% sample attrition (four parents), and the final sample size was 40 caregivers. The sample size is calculated using a test comparing two means through the Kane SP. Sample Size Calculator. ClinCalc [9].

### Tools

#### Socio-Demographic Interviewing Questionnaire

The questionnaire was developed by the researcher in Arabic after researching related literature, and it consisted of two parts. The first part contained data about the socio-demographic characteristics of family caregivers such as age, gender, marital status, and others. The second part included the socio-demographic data of children with autism, such as gender, number of siblings, and level of education.

#### The expressed emotions questionnaire

The expressed emotions questionnaire was developed by the researchers in Arabic after reviewing related literature; the researchers revised the theoretical framework and prior information in the field of expressed emotions, including several scales:

- Family Interview [10],
- Family Emotional Involvement and Criticism Scale (FEICS) [11],
- The Level of Expressed Emotion Scale Family Interview [12].

The researchers then created a new scale that was suitable for the study and its population. The subscales included five domains: hostility, criticism, over-involvement, warmth, and positive regard. These domains were represented by 47 sentences on a three-point Likert scale (always, sometimes, and never). The scoring system was designed as follows: (3) always, (2) sometimes, and (1) never. The total scores were classified as low expressed emotion if summing less than 98 and high if the score ranged from 98 to 141.

#### Parenting stress index (PSI)

Abidin *et al*. originally developed the parenting stress index [13] as a measure of parenting stress and was later modified to assess the stress faced by family caregivers of children with autism. It consists of three parts: parental distress (11 items), difficult child characteristics (11 items), and parent-child dysfunctional interactions (12 items). The scale is composed of thirty-four questions on a three-point Likert scale. The scoring system is designed as follows: (3) for agree, (2) for not sure, and (1) for disagree. Total scores are categorized as mild stress if the score ranges from 34 to 57, moderate stress if it ranges from 58 to 81, and severe stress if the score is 82 or more.

#### Reliability

The reliability of the tools was evaluated using the designed questionnaires, and the same participants were reassessed after seven days. The results were consistent across all assessments and presented high internal consistency, as revealed by the Cronbach alpha coefficient. The reliability of expressed emotions was 0.917 (excellent), while for the parenting stress scale, it was 0.983 (excellent).

#### Fieldwork

Before any data was collected, the study's purpose was conveyed to the participants. The sample was chosen based on inclusion and exclusion criteria. The data collection lasted six months, beginning in April 2022 and ending in September 2022.

Data were gathered at the outpatient clinic for autism in the pediatric hospital affiliated with Ain Shams University Hospital during morning and afternoon working hours. The researcher met with 40 family caregivers of children with autism who volunteered to take part in the study. After completing the pre-assessment, the researchers identified the needs of the family caregivers, designed sessions to address these needs, and commenced the counseling intervention sessions with the caregivers.

The counseling intervention was implemented by dividing the family caregivers into four groups, each consisting of ten caregivers. Due to the size of the clinic and the difficulty of gathering all participants at the same time, counseling interventions were introduced to each group separately. Every two weeks, each group went to the outpatient clinic on either Monday or Tuesday.

### Counseling intervention

The aim of the counseling interventions was to manage stress and reduce expressed emotions among family caregivers of children with autism. Researchers developed interventions according to caregivers' needs and relevant recent literature. The materials were written in Arabic and were divided into two parts:

#### Part I - Theoretical part

The theoretical part focused on the needs of children with autism, the family caregiver’s role in caring for a child with autism, the available services, reasons for family caregiver distress, as well always of managing stress and reducing expressed emotions.

#### Part II - Practical part

The practical part focused on providing guidance for dealing with children's difficulties, the role of family caregivers in modifying the behaviors of children with autism, effective methods for managing stress and reducing expressed emotions, improving self-esteem, managing anger, methods of expressing feelings, managing time, and practicing various relaxation techniques.

The counseling intervention consisted of 16 sessions, totaling 13 hours: four hours of theory, nine hours of practical application, and two hours of data collection and orientation. Family caregivers of children with autism were offered the skills and information needed to effectively manage stress and reduce expressed emotions by the end of the counseling session.

### Statistical Design

Using the Statistical Package for Social Sciences (SPSS) version 22 for statistical analysis of data, descriptive statistics were prepared (frequencies, percentages, mean, and standard deviation). The Pearson correlation coefficient was used for the detection of correlations between stress and expressed emotions. Chi-square analysis was used to assess the relations between variables and their characteristics.

## RESULTS

### Socio-demographic characteristics of the family caregivers

The socio-demographic characteristics show that 77.5% of the family caregivers were women, namely mothers of children with autism. Additionally, 65% were between the ages of 35 and 55, with a mean age of 40.32±3.99. Out of the participants, 92.5% were married, and 55.5% had a secondary education. Furthermore, 65% of family caregivers were working, and 80% of them resided in urban areas. Moreover, 12.5% had a positive family history of autism (Table 1).

**Table 1:** Number and percentage distribution of the sample according to their socio-demographic characteristics (n=40)

Items	No. (%)
**Consanguinity of family caregivers to the child**	
Mother	31 (77.5)
Father	9 (22.5)
**Gender** Male	9 (22.5)
Female	31 (77.5)
**Age** 20-<35	10 (25.0)
35-<55	26 (65.0)
≥ 55	4 (10.0)
Mean±SD	40.32±3.99
**Marital status** Married	37 (92.5)
Widowed	1 (2.5)
Divorced	2 (5.0)
**Educational level** No formal education	0 (0)
Basic literacy skills	2 (5.0)
Primary education	2 (5.0)
Preparatory education	6 (15.0)
Secondary education	22 (55.5)
Higher education	8 (20.0)
**Occupation** Actively working	26 (65.0)
Not actively working	14 (35.0)
**Residence** Rural	8 (20.0)
Urban	32 (80.0)
**Monthly family income** Sufficient	9 (22.5)
Fairly sufficient	18 (45.0)
Insufficient	13 (32.5)
**History of autism within the family**	5 (12.5) 35 (87.5) 3 (60.0) 2 (40.0)
Yes	
No	
**If yes, consanguinity**	
Brother	
Sister	

### Comparison between the family caregivers regarding stress

According to the result in Table 2, prior to the implementation of counseling intervention, 52.5% of the participants experienced severe parental distress, whereas this percentage decreased to 25% after the intervention. In terms of parent-child dysfunctional interaction, 47.5% of them had severe dysfunctional interaction before the counseling intervention, which decreased to 30% after the intervention. Furthermore, prior to the intervention, 70% of the studied family caregivers reported severe difficulty with their child, but this percentage decreased to 22.5% after the implementation of the counseling intervention.

**Table 2:** Comparison between the family caregivers at pre and post-intervention regarding their subscale parenting stress

Items	Pre-intervention (n=40)			Post-intervention (n=40)			X2 p-value
	Mild	Moderate	Severe	Mild	Moderate	Severe	
	No (%)	No (%)	No (%)	No (%)	No (%)	No (%)	
parental distress	5(12.5)	14(35.0)	21 (52.5)	13 (32.5)	17 (42.5)	10 (25.0)	13.561**
parent-child dysfunctional interaction	6(15.0)	15 (37.5)	19 (47.5)	12 (30.0)	16 (40.0)	12 (30.0)	10.817**
difficult child	2(5.0)	10 (25.0)	28 (70.0)	19 (47.0)	12 (30.0)	9 (22.5)	12.771**

* Significant at p<0.05. **Highly significant at p<0.01. Not significant at p>0.05 chi-square test

### Comparison between the family caregivers regarding total expressed emotions

Following the implementation of counseling intervention, a significant improvement in all domains of expressed emotions (criticism, hostility, over-involvement, warmth, and positive regard) was observed among the families, as evidenced by a p-value <0.01 (Table 3).

**Table 3:** Comparison between the family caregivers at pre and post-intervention regarding their total expressed emotions (n=40).

Total expressed emotions domains	Pre-intervention (n=40)		Post-intervention (n=40)		X2 p-value
	High	Low	High	Low	
	No (%)	No (%)	No (%)	No (%)	
The expressed emotions related to criticism	32 (80.0)	8 (20.0)	19 (47.5)	21 (52.5)	12.003**
The expressed emotions related to hostility	30 (75.0)	10 (25.0)	17 (42.5)	23 (57.5)	11.380**
The expressed emotions related to over-involvement	35 (87.5)	5 (12.5)	9 (22.5)	31 (77.5)	12.999**
The expressed emotions related to warmth	32 (80.0)	8 (20.0)	12 (30.0)	28 (70.0)	12.004**
The expressed emotions related to positive regard	30 (75.0)	10 (25.0)	11 (27.5)	29 (72.5)	11.601**
Total	32 (80.0)	8 (20.0)	13 (32.5)	27 (67.5)	13.362**

* Significant at p<0.05. **Highly significant at p<0.01. Not significant at p>0.05chi- square test

### Comparison between the family caregivers regarding total stress level

**Figure 1:**
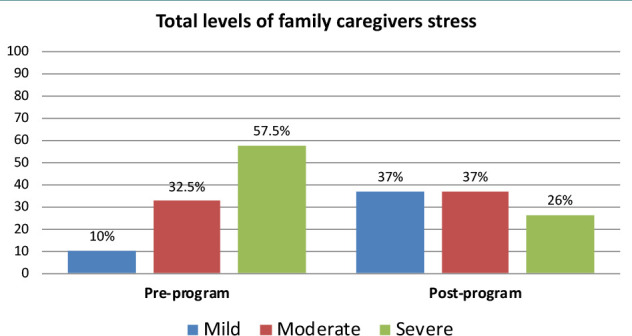
Total stress level of family caregivers pre and post-intervention (n=40)

According to Figure 1, before the implementation of counseling interventions, 57.5% of the caregivers experienced severe stress. However, after the implementation of counseling interventions, this percentage decreased to 26%.

### Correlation between study variables at pre- and post-intervention

In accordance with Table 4, there is a highly statistically significant positive correlation between total stress and total expressed emotions of family caregivers in both the pre-implementation and post-implementation stages of counseling intervention, as evidenced by p-value<0.01.

**Table 4:** Correlation between study variables at pre and post-intervention (n=40)

		Total stress	
		Pre	Post
Total Expressed Emotions	r p	0.634 0.002**	0.724 0.001**

(**) Statistically significant at p<0.01. r=Pearson correlation

## DISCUSSION

ASD is persistent neurodevelopmental affection. The families of children with autism should learn new skills to cope with their everyday challenges, as parents play a significant role in the care of children with ASD, which subjects them to significant and repeated stressors related to the children's care and the difficulty of interacting with them due to their affection. For this, counseling interventions have been recommended [14].

When comparing family caregivers at pre- and post-intervention regarding parenting stress, the results reflect that more than half of them had severe parental distress at pre-implementation of counseling intervention, compared to one-quarter at post-implementation of counseling intervention. These findings reflect the beneficial impact of the intervention on parental distress of family caregivers of children with autism who were given the necessary information and skills, such as stress management, time management strategies, and relaxation techniques.

Regarding parent-child dysfunctional interaction, the current study findings show that nearly half of the family caregivers had severe dysfunctional interaction pre-counseling, compared to less than one-third of them following the intervention. These findings reflect the beneficial effects of the counseling intervention on the parent-child interaction, which played a vital role in providing the family caregivers with the necessary information and skills about the nature of autism, the role of family caregivers, accessible resources, strategies for coping, and effective coping methods.

Concerning the difficult child, nearly three-quarters of the family caregivers had a severely difficult child at pre-counseling intervention compared to less than one-quarter of them at post-counseling intervention. These results reflect the beneficial impact of the counseling intervention on the studied population, which was necessary to provide the family caregivers with the information and skills to understand the signs and symptoms of autism and how to deal with them.

These results are supported by the study of Morsa *et al*. [15], which reported that the educational support or intervention offered by the healthcare system to people with ASD or their caregivers decreased the stress level among parents and children. Also, Gentile *et al*. [16] detected that an early intervention telehealth program may help reduce parenting stress. However, other research, such as that of MacKenzie & Eack [17], showcased no improvements in caregiving burden, family adjustment, physical health, or stress.

The present study highlighted a marked improvement in all domains of expressed emotions (criticism, hostility, over-involvement, warmth, and positive regard) among the family caregivers post-intervention (p < 0.01). These results might be due to the expressed emotions management counseling intervention sessions helping the family replace the negative emotions with positive ones and helping them learn more about the right communication skills to interact with their child in an effective way.

These results are similar to the findings of Mirzaei *et al*. [8], who detected that emotional state training improved the mother-child relationship, warmth, and positive emotions related to their children in the intervention group. Likewise, Akhani *et al*. [18] found that parent training is effective in functional emotional development and decreases negative emotions related to their children. However, this study's result disagrees with the study by Griffith *et al*. [19], who stated no significant differences in maternal emotional over-involvement or overall EE towards the child with ASD.

According to total levels of family caregiver stress, more than half of the studied family caregivers had severe parenting stress at pre-implementation of counseling intervention, compared to one-quarter of them post-implementation of counseling intervention. The findings of the present study may be related to the fact that the counseling sessions focused on time management, relaxation skills, problem-solving techniques, and ways of expressing feelings and emotions. All of these helped the caregivers manage many problems and reduce their stress.

These results are consistent with Qi *et al*. [20], who stated that psychological counseling based on problem management could effectively alleviate social anxiety and parental pressure in parents of children with ASD, improve their social support, and play a significant role in enhancing their mental health. In contrast, de Korte *et al*. [21] detected no significant improvement in parent-rated general social-communication skills; these findings justify further research on parent-group-delivered pivotal response treatment models.

Our study demonstrated a highly statistically significant positive correlation between total parenting stress and total expressed emotions of family caregivers in the pre-and post-implementation of counseling intervention. The outcome of the current study might be due to expressed emotions, and the stress of care are correlated to each other. Families who have prominent levels of anxiety, nervousness, unpleasant feelings, and expressed emotions experience cycles of sadness and dissatisfaction and perceive their caring situation as more stressful; all of these can cause a higher level of stress. These results are consistent with Seruret *et al*. [7], who reported an association between parents’ expressed emotions and their stress levels. Also, De Clercq *et al*. [22] reported that more emotional over-involvement, more criticism, and fewer expressions of warmth were associated with higher levels of parenting stress.

### Limitations

One limitation of the current research is the difficulties in organizing groups of family caregivers for the counseling session and making the necessary preparations. Additionally, ten family caregivers left the study before the counseling intervention was fully implemented, and they were replaced by new family caregivers.

## CONCLUSION

Most of the family caregivers of children with autism had elevated levels of expressed emotions, and more than half of them experienced severe levels of stress, which decreased after the implementation of counseling intervention. Additionally, there were highly statistically significant correlations between the total levels of expressed emotions and total stress levels among family caregivers during the pre-post implementation of the counseling intervention.

Based on the results of our study we propose the following recommendations: (1) periodic scientific seminars should be conducted for nurses to educate them about strategies for managing stress and methods of enhancing expressed emotions among family caregivers of children with autism, (2) psycho-educational nursing intervention programs should be periodically conducted for family caregivers to help reduce their stress levels, (3) future research focused on examining the effect of family expressed emotions on children's behavior and their overall progress and (4) further research to assess the factors that may influence the caregiving role and the many challenges faced by family caregivers in raising children with autism of different ages.
